# Modeling and Experimentation of the Unidirectional Orthodontic Force of Second Sequential Loop Orthodontic Archwire

**DOI:** 10.1155/2020/5786593

**Published:** 2020-06-11

**Authors:** Jin-Gang Jiang, Yi-Hao Chen, Lei Wang, Yong-De Zhang, Yi Liu, Wei Qian

**Affiliations:** ^1^Key Laboratory of Advanced Manufacturing and Intelligent Technology, Ministry of Education, Harbin University of Science and Technology, Harbin 150080, China; ^2^Robotics and its Engineering Research Center, Harbin University of Science and Technology, Harbin 150080, China; ^3^Peking University School of Stomatology, Beijing 100081, China

## Abstract

The abnormal tooth arrangement is one of the most common clinical features of malocclusion which is mainly caused by the tooth root compression malformation. The second sequential loop is mostly used for the adjusting of the abnormal tooth arrangement. Now, the shape devise of orthodontic archwire depends completely on the doctor's experience and patients' feedback, this practice is time-consuming, and the treatment effect is unstable. The orthodontic-force of the different parameters of the second sequence loop, including different cross-sectional parameters, material parameters, and characteristic parameters, was compared and simulated for the abnormal condition of root compression deformity. In this paper, the analysis and experimental study on the unidirectional orthodontic-force were carried out. The different parameters of the second sequential loop are analyzed, and the equivalent beam deflection theory is used to analyze the relationship between orthodontic-force and archwire parameters. Based on the structural analysis of the second sequential loop, the device for measuring orthodontic force has been designed. The orthodontic force with different structural characteristics of archwire was compared and was measured. Finally, the correction factor was developed in the unidirectional orthodontic-force forecasting model to eliminate the influence of inherent error. The average relative error rate of the theoretical results of the unidirectional orthodontic-force forecasting model is between 12.6% and 8.75%, which verifies the accuracy of the prediction model.

## 1. Introduction

Malocclusion has been regarded as one of the three major oral diseases by the World Health Organization (WHO). And the improper teeth positioning is the most common clinical features [1–6]. It usually causes the uneven tooth alignment and dental arch deformity. And it not only affects the function of mastication, but also has a certain impact on the pronunciation. The tooth decay, dentin hypersensitivity, and more mouth diseases will be accompanied by it. The incidence of malocclusion is increasing year by year in adolescents [7–11]. The second sequential loop can effectively treat the tooth root compression malformation; it pulls the misplaced teeth to the correct location through the restoring force of archwire deformation. Its therapeutic effect is related to archwire shape, cross-section, and so on [12–15].

In order to obtain accurate orthodontic force, researchers have carried out orthodontic force measurement, statics research, and finite element analysis. Shima et al. realized the calculation of bending stress by using the hyperelasticity of the archwire in the process of studying the bending properties of hollow nickel-titanium alloy wires in 2010 [16]. The measurement and research of orthodontic force are mainly carried out in the laboratory environment. The archwire placed on the dental model is used to measure the force, which avoids the limitation of the space in the oral cavity and ensures the safety of patients [17–20]. Kinzinger et al. designed a pendulum force measuring device, which simulated the oral environment and measured the effect of orthodontic force on the crown and root in 2004 [21]. Fuck and Drescher is based on a six-dimensional force sensor to measure the plaster dentition model in 2005. The sensor is connected with the crown. The position and posture of the model can be regulated freely [22, 23]. Lapatki et al. planed an integrated bracket, which integrates the pressure pickup into the bracket and can directly measure the pressure between the archwire and the bracket, but it cannot truly reflect the actual relationship between the archwire and the bracket in the clinical state [24]. Badawi et al. have established a measuring device with a three-dimensional orthodontic force in 2010, which replaces the actual teeth with a simplified cylinder. It can preliminarily measure the orthodontic force of all the teeth on the dental arch and distinguishes the difference between passive ligation and other ligation methods [25]. Chen et al. added a T-type loop to the dental moulds of the first premolar and incisor extraction in 2010 and analyzed the effect of orthodontic force produced by the T-type loop on two teeth that are in contact with each other [26]. Wei et al. designed the measure model of archwire and bracket in 2012. By discussing the influence of archwire features on the orthodontic force system, a math means for assessing the force system in orthodontic treatment was proposed [27]. Mencattelli et al. built a device to measure the force of the teeth under various orthodontic conditions in 2015. Taking the dental plaster model as the research object, the force of teeth under four kinds of hyperelastic archwire and two kinds of invisible orthodontic appliances was analyzed [28]. In 2016, Midorikawa et al. designed a set of orthodontic force sensing system, which can measure the stress state of 14 teeth at the same time [29]. In 2017, Higa et al. constructed a stress measuring device for small-diameter nickel-titanium alloy wires to measure the force produced by the wires. The mechanical characteristics of the traditional nickel-titanium alloy archwire and thermally activated nickel-titanium alloy wires under different ligation modes were studied. The results show that the traditional nickel-titanium alloy wires release higher orthodontic force [30]. Lai et al. made a dental model with resin and simulated the temperature of the oral environment with a heating rod in 2018. Based on this, a force analysis system for the archwire was established [31]. Scholars have analyzed and measured the orthodontic force by means of experimental simulation and mechanical analysis through the discussion of research status. Scholars gain the orthodontic force by measuring the orthodontic force on the teeth, but the effective orthodontic force is delivered by the stent. Therefore, in order to obtain accurate measurement results, orthodontic archwires are directly measured in orthodontic force sources. Much as the relationship between the features of the archwire and the orthodontic force is considered. The relationship between archwire bending features and orthodontic force is invalid. These researches cannot guide doctors to accurately speculate the orthodontic force produced by designing archwire [10].

This study carries out the experiment and modeling of the second sequential loop to help doctors handle the problem of adjusting excessive and small orthodontic force by experience, which may cause secondary injury to patients and slowing down the treatment time. Firstly, the extraoral measurement method and orthodontic force measurement device based on a six-dimensional force sensor are designed, and the straining characteristics of the second sequence loop are analyzed. Secondly, the different parameters of the second sequence loop, including different cross-sectional parameters, material parameters, and characteristic parameters, were compared and simulated for the abnormal condition of root compression deformity. Finally, the orthodontic force forecasting model is established after the error compensation. The establishment of the model can provide a quantitative calculation for orthodontic treatment. The model predicts the relationship between the features of the orthodontic archwire and the orthodontic force. It can help doctors design customized orthodontic archwire and improve the scientificity of the orthodontic treatment. And it can reduce patients' pain and improve treatment efficiency.

## 2. Methods

### 2.1. Orthodontic-Force Loading Unit of the Second Sequential Loop

**Figure 1:**
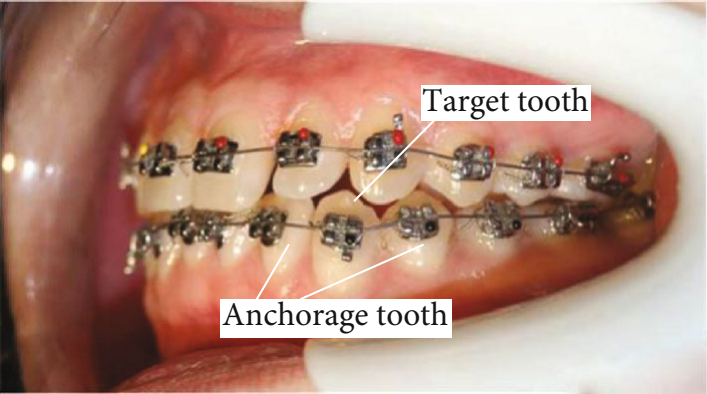
Second sequential loop.

Figure 1 shows the basic loading units of orthodontic force, which are the functional archwire and the sequential loop. The most important factor affecting the orthodontic force is the shape of orthodontic archwire. In this paper, the orthodontic-force is measured and quantitatively analyzed with sequence curvature as the research object.

The sequence loop is able to be classified into three types based on the shape and the function of the archwire, which are the first, the second, and the third sequential loop. The arch form of the deformed maxillary and mandibular after the treatment of the first sequential loop is the same as the natural shape. In the direction of buccal-lingual, the first sequential loop can be used to treat the light dislocation. However, when the dislocation is serious, we will consider to combine the first sequential loop with the functional loop. The posterior teeth and the anterior ones are raised and depressed, when the tip forward curve of the second sequential loop only bents in the vertical plane. Furthermore, the third sequential loop is mainly utilized to generate the torque to move the tooth root and tongue side, because it is able to only be bent by the square section archwire.

The main parameters in this paper are as follows: *F* is the orthodontic force; *M* stands for the material characteristics of the archwire; *S* represents the section characteristics of the archwire; and *P* is the archwire's feature parameters. Stainless steel archwire, Australian archwire as the most commonly used orthodontic archwire.

Furthermore, the characteristic parameters of the archwire are as follows: *E* is a symbol of modulus of elasticity; the Poisson's ratio is *μ*; *S*_*A*_ and *S*_*S*_ represent sectional area and sectional shape of archwire's section characteristics; *I* stands for the inertia moment to the bending axis. The type of archwire curve decides the characteristic parameter of the archwire.

Thus, the basic form of the prediction model of the orthodontic force is as follows.

(1) $$\begin{array}{c} F=F\left(M,S,P\right). \end{array}$$

### 2.2. Unidirectional Orthodontic-Force Prediction Modeling

The orthodontic force is affected by many factors in its generation process due to the complexity of the orthodontic treatment. Therefore, these main parameters should be considered to establish the unidirectional orthodontic force forecasting model, and the related experiments and experimental results are designed and analyzed to correct this forecasting model. Thus, the basic assumption had been developed as follows to simplify the establishment process of the prediction model:

1. The orthodontic archwire and the orthodontic brackets are ligated tightly, which means the sliding friction is not existing between the orthodontic archwire and the orthodontic brackets
2. The orthodontic force generated on the tooth is the restoring force generated by the elastic deformation of the orthodontic archwire

**Figure 2:**
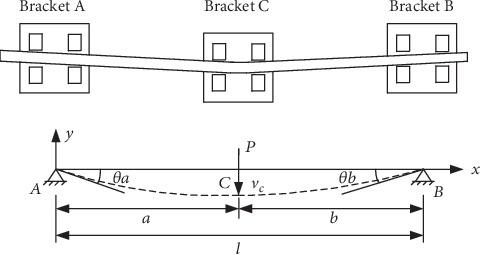
The force diagram of root compressing deformity.

Because of the representativeness of the second sequential loop, its orthodontic-force forecasting model will be studied in this paper. The tooth compression generated in the root direction may lower the tooth. After the archwire is inserted into the bracket, the orthodontic force on the root cap direction will be generated on the target tooth, and the anchorage is the adjacent teeth.  Figure 2 shows the connection relation between the bracket and the archwire.

According to the materials of mechanics, this model is simplified as a simply supported beam model [10], and the axis of the beam is *x*-axis. The deflection curve differential equation is given as follows:

(2) $$\begin{array}{c} \frac{{\text{d}}^{2}v}{\text{d}x^{2}}=\frac{M\left(x\right)}{{EI}_{z}}. \end{array}$$

where *v* represents the horizontal arm's moving distance and the vertical arm's bending deflection as well. *M*(*x*) stands for the *x*-axis bending moment of the vertical arm, *E* is the modulus of elasticity of the archwire, and *I*_*z*_ is the inertia moment generated by the archwire section to the *z*-axis. For the round-section archwire, the inertia moment is *I*_*z*_ = *π*(2*r*)^4^/64, where *r* is the radius of the round section. For the rectangular section archwire, the inertia moment is *I*_*z*_ = *c*_1_*c*_2_^3^/12, where *c*_2_ is the side length parallel to the *z*-axis, and *c*_1_ is the side length perpendicular to the *z*-axis [32].

The angular equation is represented by *θ*(*x*), and*v*(*x*) stands for the deflection equation. Then, the second sequential loop is able to be represented via the integral of Equation (2).

(3) $$\begin{array}{c} \theta \left(x\right)=\frac{\text{d}v}{\text{d}x}=\int \frac{M\left(x\right)}{{EI}_{\text{z}}}\text{d}x+C, \end{array}$$

(4) $$\begin{array}{c} v\left(x\right)=\int \int \frac{M\left(x\right)}{{EI}_{z}}\text{d}x\text{d}x+Cx+D, \end{array}$$

where *C* and *D* are constants of the integration which are decided by the condition of boundary and continuity [32]. The bending moment equation of the second sequential loop is as follows.

(5) $$\begin{array}{c} \left\{\begin{array}{c} M\left(x\right)=\frac{Pb}{l}x,\left(0\leq x\leq a\right),\\ M\left(x\right)=\frac{Pb}{l}x-P\left(x-a\right),\left(a\leq x\leq l\right), \end{array}\right. \end{array}$$

where *l* represents the length of the archwire between the two anchorage teeth which named as the anchorage distance. *a* is the distance between the target tooth and the distal anchorage tooth which named as the offset distance [12]. The distance between the target tooth and the mesial anchorage tooth is represented by *b*.

Substituting Equations (2) and (5) into Equations (3) and (4), respectively, the following equations can be obtained.

(6) $$\begin{array}{c} \left\{\begin{array}{c} \theta \left(x\right)=\frac{Pb}{l}\frac{x^{2}}{2}+C_{1},\left(0\leq x\leq a\right),\\ \theta \left(x\right)=\frac{Pb}{l}\frac{x^{2}}{2}-P\frac{{\left(x-a\right)}^{2}}{2}+C_{2},\left(a\leq x\leq l\right), \end{array}\right. \end{array}$$

(7) $$\begin{array}{c} \left\{\begin{array}{c} v\left(x\right)=\frac{Pb}{l}\frac{x^{3}}{6}+C_{1}x+D_{1},\left(0\leq x\leq a\right),\\ v\left(x\right)=\frac{Pb}{l}\frac{x^{3}}{6}-P\frac{{\left(x-a\right)}^{3}}{6}+C_{2}x+D_{2},\left(a\leq x\leq l\right). \end{array}\right. \end{array}$$

*C*_1_, *C*_2_, *D*_1_, and *D*_2_ can be determined by the boundary condition and continuity condition. Hence, *C*_1_ = *C*_2_ = ‐(*Pb*/*l*)(*l*^2^ − *b*^2^), *D*_1_ = *D*_2_ = 0.

Substituting *C*_1_, *C*_2_, *D*_1_, and *D*_2_ into Equations (7), the following equations can be obtained.

(8) $$\begin{array}{c} \left\{\begin{array}{c} v\left(x\right)=-\frac{Pbx}{6{EI}_{z}}\left(l^{2}-x^{2}-b^{2}\right),\left(0\leq x\leq a\right),\\ v\left(x\right)=-\frac{Pb}{6{EI}_{z}}\left[\frac{l}{b}{\left(x-a\right)}^{3}+\left(l^{2}-b^{2}\right)x-x^{3}\right],\left(a\leq x\leq l\right). \end{array}\right. \end{array}$$

Therefore, the *v* on the position *x* = *a* affected by the orthodontic-force is able to be expressed as follows:

(9) $$\begin{array}{c} v=-\frac{Pa}{6EI}\left(l^{2}-a^{2}-b^{2}\right) \end{array}$$

The orthodontic force *F* is the counter force of the acting force (*P*) which generated the deformation based on the principle of acting force and the counter force [32].

(10) $$\begin{array}{c} F=-P=\frac{6EId}{a\left(l^{2}-a^{2}-b^{2}\right)}, \end{array}$$

where *d* is the moving distance of the archwire in the *y* direction.

## 3. Results

### 3.1. Design of the Measuring Method of the Second Sequential Loop Unidirectional Orthodontic-Force

**Figure 3:**
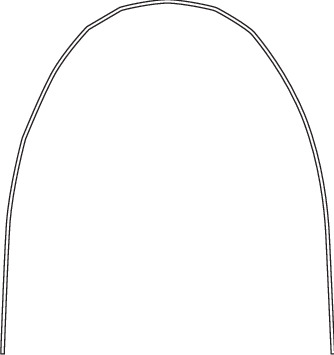
Standard oval loop.

Sequential loop is one of the basic loading units of orthodontic-force [33–35]. The archwire is needed to be bent into the shape of a standard arch, which its initial shape is straight. After the archwire is fixed with the bracket, the preformed orthodontic archwire will be deformed to generate the orthodontic force because of the limitation of the bracket position. For the purpose of bending the archwire to fit the dentition shape of the patient, it is necessary to adjust the shape of the standard archwire. The orthodontic doctors always bend the first, the second, and third sequential loop to adjust the shape of the standard archwire. According to the standard arch map of the mandible, the preformed egg shape archwire of the mandibular is shown in Figure 3.

**Figure 4:**
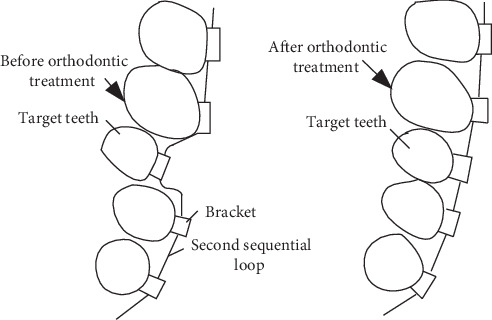
Simulation of tooth moving process affected by the second sequential loop.

The deformation of the second sequential loop generated the orthodontic-force. The amount and type of deformation is the determining of the orthodontic force. During the actual orthodontic treatment, the patient had worn the standard archwire on his misplaced teeth. Thus, the deformation will be generated on the archwire at the misplaced teeth. And the deformation may generate restoring force. Due to the influence of the restoring force, the misplaced teeth are driven to the standard position. Thus, it is difficult to measure the orthodontic force in the complex force deliver process. In this paper, we consider the extraoral measurement method and use the loading unit to generate the displacement load on the standard archwire. The displacement load drives the target tooth to the abnormal position to simulate the orthodontic process [12]. In order to facilitate subsequent research, the six-dimensional force sensor is used to measure the magnitude of the orthodontic force. The six-dimensional force sensor can simultaneously detect the three-dimensional full force information, that is, three force components and three-moment components. The detection of all the information at the same time can eliminate experimental interference and conform to the principle of a single variable. As shown in Figure 4, the measured-force of different displacement load is the orthodontic-force.

### 3.2. Design of the Measuring Device of the Second Sequential Loop Unidirectional Orthodontic-Force

**Figure 5:**
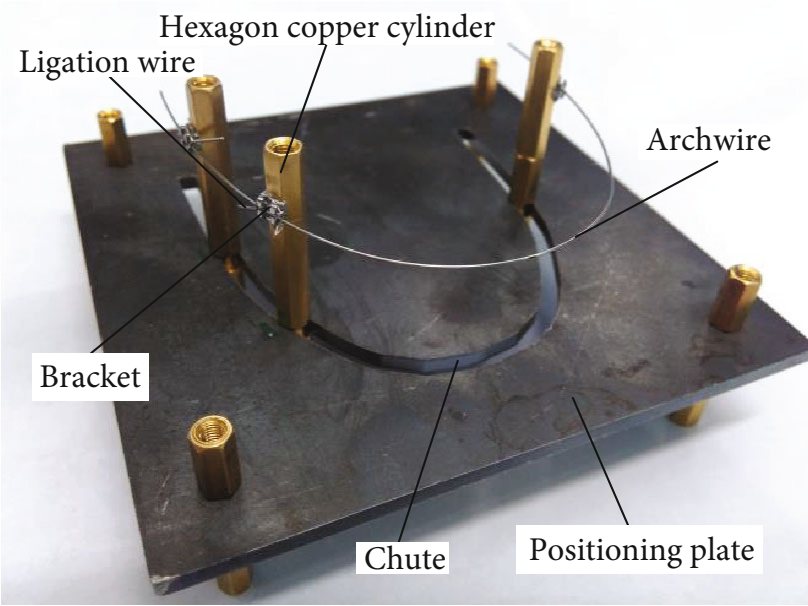
Simulative orthodontic environment.

In order to avoid the patient' hurt, in this paper, we use an “in vitro” measurement due to the narrow space of the human mouth. The positioning plate is fixed on the table, the hexagonal stud simulates teeth, the chute simulates human gums, and the friction between the chute track, and hexagonal stud is equivalent to the resistance in the orthodontic process. The orthodontic archwire and bracket are installed according to the real orthodontic process, especially the ligation wire adopts the way of tight ligation, that is, the sequential loop archwire will not generate sliding friction in the bracket. The position of the archwire was fixed according to the actual tying method to simulate the real in-mouth working environment, which is shown in Figure 5.

The design of sequential curve measuring device should meet the following requirements.

1. The measuring device is required to meet the measurement requirements of the six-dimensional force. The force in one-direction can be measured separately, and the superposition of force and force in multiple directions can be measured simultaneously
2. The adjustment accuracy of the straight movement of the measuring device should be 0.1 mm. The setting range should be more than 5 mm. The adjustment accuracy of the rotation should be 1°. The setting range should be more than 10°
3. The measuring range of the force measuring device should be greater than the maximum amount of orthodontic-force that can be applied to the sequential loop. The measuring range should not be less than 15 N or 50 N•mm, and the resolution should not be less than 0.1 N or 0.01 N•mm

According to the measurement method and requirement, the installation way of measuring devices is as follows. The arched chute is made on the positioning plate. The shape of the arched chute is the same as the standard arch type. The hexagon copper cylinders are mounted on the arched chute to simulate the position of the actual teeth. The locations of the hexagon copper cylinders can be adjusted along the arched chute. The brackets are pasted on the hexagon copper cylinders. The archwire is fixed on the brackets by the clinical ligation mode [12].

**Figure 6:**
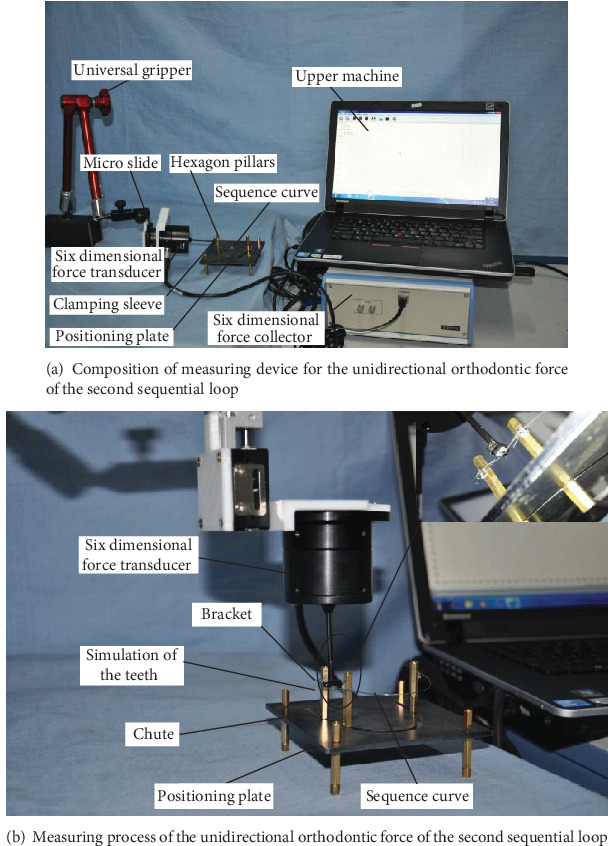
Measuring device for the unidirectional orthodontic force of the second sequential loop.

Two hexagonal copper pillars can simulate two nonadjacent teeth at any tooth position, and their distance is equivalent to the anchorage distance. The position of the sensor needs to be adjusted flexibly to adapt to different measuring positions. When measuring the orthodontic-force, a six-dimensional force sensor is installed on the adjusting device. When measuring the unidirectional orthodontic-force, a micro moving sliding table is used as a displacement adjustment device to adjust the distance from the crown to the root, as shown in Figure 6.

Adjusting device through the fittings installed, in the end of the execution of the universal holder by universal gripper driven sensor positioning at any position. The sensor through the clamping sleeve clamp under test, the archwire after the positioning of the sensor, by adjusting the micro sliding table-driven clamping set of movement, to exert the archwire displacement load, its deformation is equal to the offset distance.

The correction force is collected by the six-dimensional force collector and sent to the upper computer for display and subsequent processing.

### 3.3. Second Sequential Loop Unidirectional Orthodontic-Force Measuring Experiment

The unilateral orthodontic forces of the typical root and the buccal tongue were measured by the second sequential loop measurement device; measured objects were the standard preformed egg shape archwire. In the measurement of the same type of archwire, the manual measurement error is less than 5%. In each experimental measurement, three the same types of archwires were utilized to conduct three groups' repeated experiments. The effective measurement data of one type of the archwire is calculated by averaging the three measurement values.

In this experiment, the naming method of the used sequential loop can refer to Reference [10]. For example, S16162010 indicates that the size of the cross-section of stainless steel square archwire is 0.016 × 0.016 inch, the anchorage distance is 20 mm, and the offset distance is 10 mm.

The orthodontic measuring experiment is conducted according to the loading characteristics of the tooth root compression malformation. The moving distance (Preset tooth deformity) is limited to 5 mm, we divided the range of 5 mm into 10 measuring points, and each point is the hiatus of 0.5 mm for measuring the orthodontic-force of 10 points.

We use the method of controlling a single parameter to study the effects on orthodontic-force, which is caused by the different anchorage distances, different moving distances, different offset distances, different cross-section characteristics, and different material properties, ten different types of the archwires are used. The second sequential loops used in the experiment are listed as follows. ①-S16162010; ②-S16162210; ③-S16162410; ④-S16162610; ⑤-S16162009; ⑥-S16162008; ⑦-S20102007; ⑧-S18252010; ⑨-S00162010; ⑩-A16162010. The measuring results are listed in Table 1.

**Figure 7:**
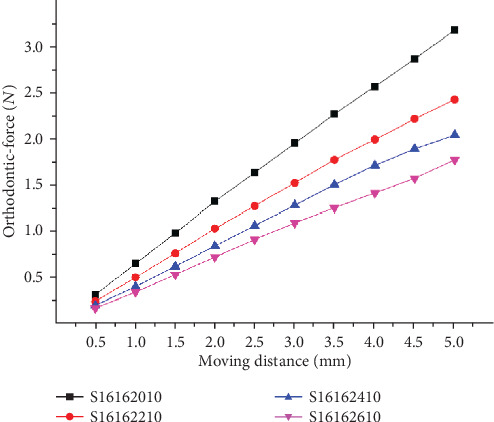
Orthodontic-force at different anchorage distances.

Figure 7 showed the experimental results. The orthodontic force has a negative connection with the anchorage distances. The orthodontic force decreases when the anchorage distances become greater. Thus, a longer anchorage distance would be expected to generate a mild orthodontic force.

**Figure 8:**
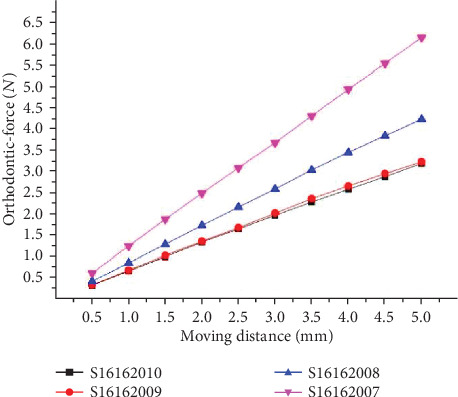
Orthodontic-force at different offset distances.

The orthodontic-force has a negative connection with the offset distance through comparison result of ①, ⑤, ⑥, and ⑦. The orthodontic-force increases when the offset distances become smaller. The comparison result is as shown in Figure 8.

**Figure 9:**
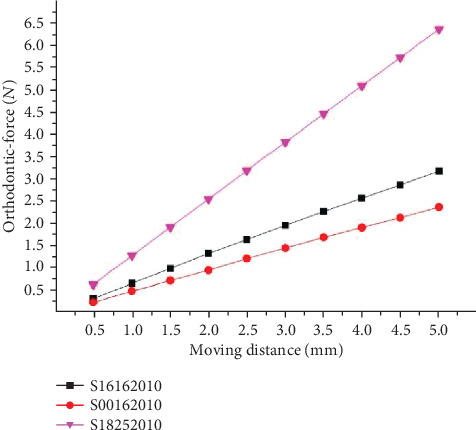
Orthodontic-force at different sections.

As shown in Figure 9, through the comparison result of ①, ⑧, and ⑨, the load-deflection rate of round archwire is lower than the square archwire. The load-deflection rate of the larger section of the square archwire is higher than the small section. The orthodontic-force is has a positive connection with the moment of inertia.

**Figure 10:**
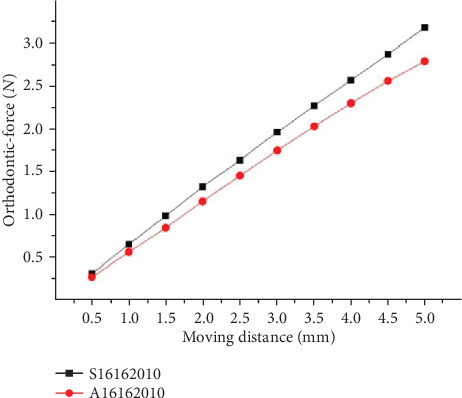
Orthodontic-force with different materials.

As shown in Figure 10, the load-deflection rate of the Australian archwire is lower than that of the stainless steel archwire, and the orthodontic-force is obviously weakened. The orthodontic-force has a positive connection with the elastic modulus of the archwire.

It can be seen from the prediction model of the unidirectional orthodontic-force that the orthodontic-force is proportional to the moving distance, the moment of inertia, and the elastic modulus and inversely proportional to the cubic function of the anchorage distance and the quadratic function of the offset distance. Therefore, it is inversely proportional to the moving distance of the sequence loop. The effect of each parameter on the orthodontic-force is the same as that reflected in the sequence prediction model.

## 4. Discussion

In the acting process of the orthodontic archwire, the friction force may be also generated because of the cooperation of the archwire and the brackets. This part cannot be ignored. And the archwire is made by the manual and robotic bending process, and the residual stress could be generated on the bent archwire because of the deformation of the bent archwire. These factors could influence the establishment of the prediction in this study which may generate the difference between the experimental result and the theoretical result. To solve this problem, the correction factor, *K_F_*, is developed to lower the deviation rate between the orthodontic-force prediction model and the experimental result. By comparing the experimental result with the theoretical result, *K_F_* can be calculated through the following equation.

(11) $$\begin{array}{c} F=\frac{6{EIdK}_{F}}{a\left(l^{2}-a^{2}-b^{2}\right)}. \end{array}$$

There two important parameters in the following analysis of the experimental result, which are theoretical deviation rate and theoretical correction rate. Firstly, the theoretical deviation rate can be obtained through the ratio calculating between the difference, which is calculated through the subtraction of the experimental and the theoretical value obtained without developing the correction factor and the experimental value. Secondly, the theoretical correction rate can be calculated through the ration calculating between the difference which is calculated through the subtraction of the experimental results and the theoretical data without developing the correction factor and the theoretical value. The correctness of the prediction model without considering the correction model compared with the experimental result can be shown through the calculating of the theoretical deviation rate. And the correcting influence of the correction factor generated to the prediction model can also be shown through the calculating of the theoretical correction rate. The theoretical value calculated without developing the correction factor is drawn in solid lines, and the theoretical deviation rate is drawn in the dotted lines.

**Figure 11:**
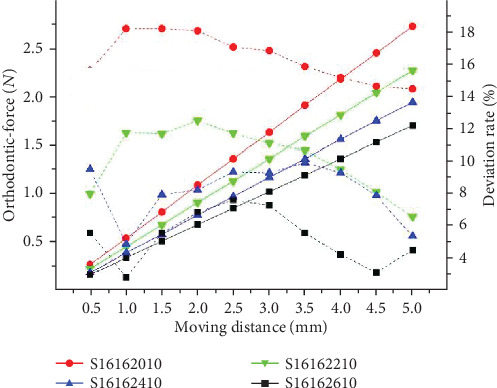
The results of theoretical calculations of orthodontic force under different anchorage distance.

The comparison experiment between the sequential loop bent by the same archwire but the different anchorage distances are conducted through the archwires coded as ①, ②, ③, and ④. Through the results shown in Figure 11, it can be found that the orthodontic force caused by the sequential is negatively related to anchorage distance. The theoretical deviation rate is also a negatively relationship with the anchorage distance.

Through the fitting of the theoretical correction rate of different anchorage, theoretical correction coefficient affected by anchorage *K*_*Fa*_ can be obtained as follows:

(12) $$\begin{array}{c} K_{Fl}=\frac{1+\left(-2.2245l\times 10^{3}+62.561\right)}{100}. \end{array}$$

**Figure 12:**
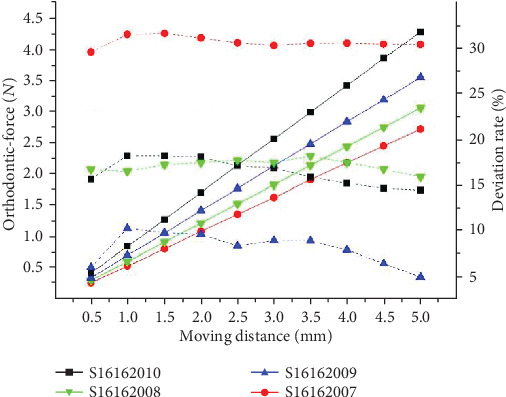
The results of theoretical calculations of orthodontic-force under different offset distance.

The comparison experiment between the sequential loop bent by the same archwire but the different offset distances are conducted with the archwire shown in Figure 12. Through the comparison between its theoretical and experimental result, it can be found that the orthodontic force caused by the sequential is negatively related to offset distance. The theoretical deviation rate is also negatively related to the anchorage distance in a quadratic function.

The theoretical correction coefficient of the influence of the offset distance is:

(13) $$\begin{array}{c} K_{Fa}=\frac{1+\left[8.555{\left(a\times 10^{3}\right)}^{2}-153.941a\times 10^{3}+703.031\right]}{100}. \end{array}$$

**Figure 13:**
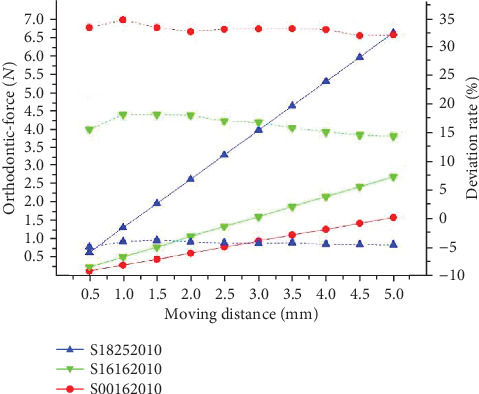
The results of theoretical calculations of orthodontic force under different sections.

The comparison experiment between the sequential loop bent by the different archwires but the same bending parameters are conducted through the archwires coded as ①, ⑧, and ⑨.  Figure 13 showed these results; therefore, we found that the theoretical deviation rate is negatively related to the moment of inertia of the section.

Through the fitting of the theoretical correction rate of different cross-sections, the theoretical correction coefficient affected by different cross-sections *K*_*Fs*_ can be obtained as follows:

(14) $$\begin{array}{c} K_{FS}=\frac{1+\left[‐36.5\ln\left(I\right)+53.706\right]\times 10^{15}}{100}. \end{array}$$

**Figure 14:**
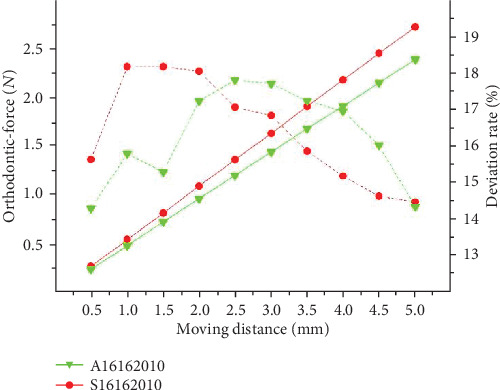
The results of theoretical calculations of orthodontic force under different materials.

The comparison experiment between sequential loop bent by the archwires with different materials but the same bending parameters are conducted through the archwires coded as ① and ⑩. Through the results shown in Figure 14, we found that orthodontic force is actively relationship with elastic modulus. The theoretical deviation of orthodontic-force is negatively related to the elastic modulus of materials.

According to the fitting of the theoretical correction rate of different materials, the theoretical correction coefficient affected by different materials *K*_*FM*_ can be obtained as follows:

(15) $$\begin{array}{c} K_{FM}=\frac{1+\left(-0.1068E\times 10^{10}+20.5699\right)}{100}. \end{array}$$

Through the analysis above, the correction factors *K*_*F*_ and *K*_*M*_ could be affected by the anchorage distance, offset distance, and the material. Multiplying the correction factors directly may over correcting the prediction model. However, the archwire coded as S16162010 is used in every calculating process of the correction factors of each parameter. Thus, the correction factor of the S16162010 can be used as a base parameter to calculate the general correction parameter of the prediction model. The unidirectional orthodontic-force correction coefficient *K*_*F*_ can be obtained as follows:

(16) $$\begin{array}{c} K_{F}=K_{Fl}\cdot \frac{K_{Fa}}{K_{Fa0}}\cdot \frac{K_{FS}}{K_{FS0}}\cdot \frac{K_{FM}}{K_{FM0}}. \end{array}$$

Then, the prediction model for the orthodontic force of the sequential loop with the correction factor is as follows:

(17) $$\begin{array}{c} F=K_{Fl}\cdot \frac{K_{Fa}}{K_{Fa0}}\cdot \frac{K_{FS}}{K_{FS0}}\cdot \frac{K_{FM}}{K_{FM0}}\cdot \frac{6EIdK}{a\left(l^{2}-a^{2}-b^{2}\right)}. \end{array}$$

**Figure 15:**
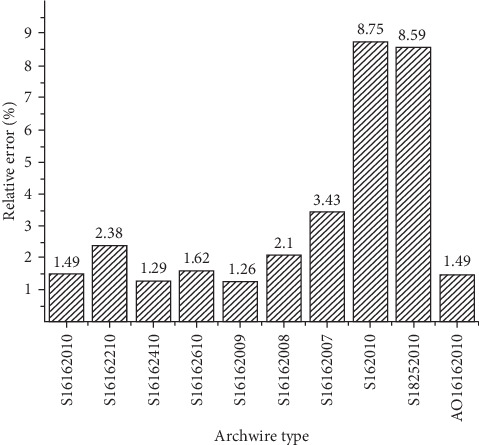
Relative error rate calculated by the theoretical calculation of sequential loop orthodontic force prediction model

Figure 15 showed that the error rate between the prediction model theoretical data and the experimental value. The relative error rate of the sequential loop orthodontic force prediction loop was calculated and it is ranging from 1.26% to 8.75%. It filled with the requirement of the prediction of the orthodontic force. Orthodontic doctors can use the prediction model to calculate the orthodontic force through the bent archwire.

## 5. Conclusions

The selection of orthodontic archwire shape is determined by doctors, which based on doctors' clinical experience and actual experience of the patient in orthodontic treatment. It cannot ensure the treatment effectiveness and the patient's comfort. Therefore, the prediction model of unidirectional orthodontic-force was built in this study to help doctors understand the method of quantifying the orthodontic force, which is generated by the second sequential loop according to known parameters, for example, moving distance and shape of the material section. The second sequential loop was studied as the basic loading unit. The characteristic parameters, material parameters, and cross-section parameters of the second sequential loop were used as the valid parameters. The second sequential loop orthodontic-force measuring equipment is designed according to the orthodontic force transmission characteristics and is equipped with a six-dimensional force sensor. Furthermore, the measuring experiments were performed on the second sequential loops of various parameters. The comparison of the prediction model theoretical parameters and the experimental result was proposed to calculate the orthodontic force influencing factor and eliminate the errors of the model. Finally, according to established the orthodontic force influencing factor, the prediction model of the second sequential loop was built. The average relative error rate of the prediction model was ranging from 1.26% to 8.75%, thus, the experiment result can satisfy the requirements of orthodontic force prediction. The model can help doctors design customized orthodontic archwire and improve the scientificity of orthodontic treatment. And it can reduce patients' pain and improve treatment efficiency.

## Figures and Tables

**Table 1:** The unidirectional orthodontic force measuring results of different second sequential loops (*N*).

Moving distance (mm)	Type of archwire									
	I	II	III	IV	V	VI	VII	VII	IX	X
0.5	0.32	0.25	0.21	0.18	0.33	0.42	0.61	0.63	0.24	0.28
1.0	0.66	0.51	0.41	0.35	0.68	0.85	1.24	1.28	0.49	0.57
1.5	0.99	0.77	0.63	0.54	1.02	1.28	1.87	1.92	0.72	0.85
2.0	1.33	1.04	0.85	0.73	1.35	1.72	2.48	2.55	0.95	1.16
2.5	1.64	1.28	1.07	0.92	1.67	2.15	3.08	3.19	1.21	1.46
3.0	1.96	1.53	1.29	1.10	2.01	2.58	3.67	3.82	1.45	1.75
3.5	2.27	1.78	1.51	1.26	2.35	3.03	4.30	4.46	1.69	2.03
4.0	2.57	2.00	1.72	1.42	2.65	3.44	4.92	5.09	1.91	2.3
4.5	2.87	2.22	1.90	1.58	2.94	3.83	5.53	5.72	2.13	2.56
5.0	3.18	2.43	2.05	1.78	3.22	4.22	6.13	6.35	2.37	2.79

## Data Availability

The data that support the findings of this study are available on request from the corresponding author, Jingang Jiang.
